# P-494. Machine learning for detection of Rising Trends in Perinatal HIV Transmission Risk: A five-Year Analysis with a 2024 Surge

**DOI:** 10.1093/ofid/ofaf695.709

**Published:** 2026-01-11

**Authors:** Morouge M Alramadhan, Gilhen Rodriguez, Hassan S Al Khatib, Perez Norma, James Murphy, Gloria P Heresi

**Affiliations:** University of texas Health and science at Houston, Houston, TX; University of Texas McGovern Medical School, Houston, Texas; Mississippi State University, Houston, Texas; University of Texas Health science center at Houston/ McGovern Medical School, Houston, Texas; UTHealth, McGovern Medical School, Houston, Texas; University of texas Health and science at Houston, Houston, TX

## Abstract

**Background:**

In 2024, our perinatal HIV consultation service encountered a precipitous increase in babies at high risk of HIV infection (Figure 1) and our objective to utilize machine learning (ML) methodologies to identify predictors, causal inferences, and points of intervention.

**Figure:**
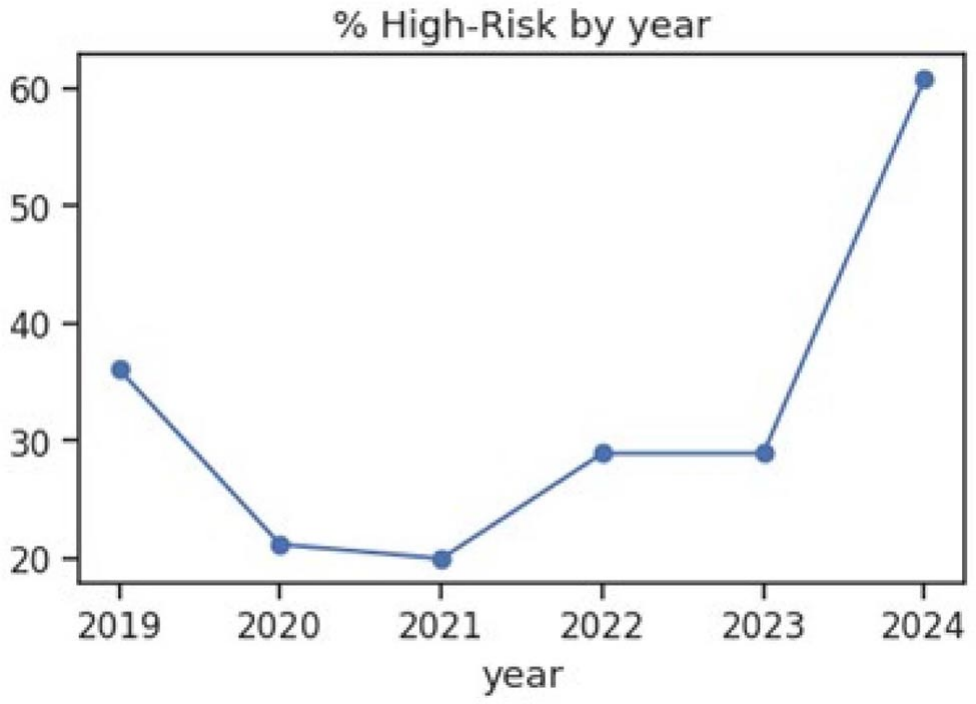
% High-Risk by year

**Figure:**
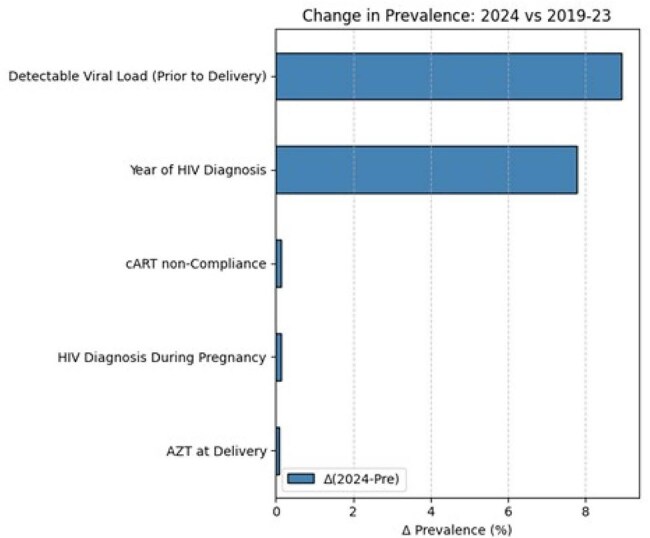
Change in Prevalence:2024 vs 2019-2023

**Methods:**

216 deliveries (2019-2024) from HIV positive mothers were reviewed. The newborn was classified as high risk if: acute HIV infection occurred during pregnancy, HIV viral load was >50 RNA copies/ml within 4 weeks of delivery, or there was poor adherence to ART. Data were from the EMR.

Influential predictors were identified using gradient-boosted trees with Shapley Additive Explanations value interpretation. Causal inference techniques estimated treatment effects while adjusting for confounders. Prevalence shift and interaction models examined year-over-year trends. Decomposition and counterfactual simulations also estimated contribution of variable prevalence changes to observed trends.

**Results:**

High-risk infants increased from 27.6% (2019-2023) to 60.9% in 2024. Key determinants: delayed ART initiation (48% increased risk), detectable viral load (65%), and ART non-compliance (interaction coefficient 1.83, p 0.032). The 2024 cohort exhibited higher rates of detectable viral loads (+8.9 units, p< 0.001) and earlier HIV diagnosis (+7.8 units, p< 0.001), i.e., on average, women delivering in 2024 had been diagnosed with HIV 7.8 years more recently compared to women who delivered in earlier years as shown in Fig. 2. Statistical decomposition revealed that 57.8% of increased high-risk cases were attributable to shifts in patient profiles, while 42.2% resulted from enhanced effects of existing risk factors. Counterfactual analyses demonstrated that reverting viral load suppression and ART initiation patterns to 2019-2023 levels would have mitigated approximately 26% of the observed spike (Figure 2).

**Conclusion:**

Using EMR data, our advanced analytics methods attributed the increase in high-risk infants to a rise in higher-risk mothers rather than changes in clinical standards and identified intervention targets. Importantly, the methodology is sensitive to embedding within the EMR matrix, hopefully enabling a rapid, automated method to identify trends, causations, and points of intervention.

**Disclosures:**

All Authors: No reported disclosures

